# Hydrothermally Synthesized Mg-Based Spinel Nanoferrites: Phase Formation and Study on Magnetic Features and Microwave Characteristics

**DOI:** 10.3390/ma11112274

**Published:** 2018-11-14

**Authors:** Chien-Yie Tsay, Yi-Chun Chiu, Chien-Ming Lei

**Affiliations:** 1 Department of Materials Science and Engineering, Feng Chia University, Taichung 40724, Taiwan; a0928402248@gmail.com; 2 Department of Chemical and Materials Engineering, Chinese Culture University, Taipei 11114, Taiwan; ljm9@faculty.pccu.edu.tw

**Keywords:** magnesium ferrite, nanoparticles, hydrothermal synthesis, magnetic properties, superparamagnetism, microwave properties

## Abstract

Three kinds of magnesium-based spinel nanoferrites with the chemical formulas of MgFe_2_O_4_ (Mg ferrite), Mg_0.9_Mn_0.1_Fe_2_O_4_ (Mg-Mn ferrite), and Mg_0.9_Mn_0.1_In_0.1_Fe_1.9_O_4_ (Mg-Mn-In ferrite) were synthesized by hydrothermal route. We report the composition-dependent magnetic parameters and microwave properties of Mg-based ferrite nanoparticles. XRD results revealed that the Mg-based ferrite nanoparticles exhibited a cubic spinel structure and had an average nanocrystallite size in the range of 5.8–2.6 nm. Raman spectroscopy analysis confirmed the formation of cubic-spinel phase Mg-based nanoferrites. The room-temperature magnetization measurements indicated that the Mg ferrite nanoparticles had superparamagnetic behavior; whereas the Mg-Mn and Mg-Mn-In ferrite nanoparticles exhibited a paramagnetic nature. The microwave properties of obtained ferrite nanoparticles were studied by alternating current (AC) magnetic susceptibility measurement and electron paramagnetic resonance (EPR) spectroscopy. It was found that the un-substituted Mg ferrite sample exhibited microwave characteristics better than those of the Mn substituted and Mn-In co-substituted Mg ferrite samples.

## 1. Introduction

Nano-sized ferrite particles have attracted scientific interest and been widely explored because they show specific features such as the quantum size effect, the magnetic tunneling effect, and superparamagnetic behavior. These magnetic oxide nanoparticles have an important role in the fields of energy storage, environmental protection, biomedicine, nanoelectronics, and communication technology due to their ease of manufacture, chemical stability, high surface-to-volume ratio (size effect), appropriate magnetic properties, relatively non-toxic nature (being environmentally-friendly), and biocompatibility [1,2,3]. Mg ferrite (MgFeO_4_), which has a spinel crystal structure, is a soft magnetic semiconducting material and has very high electrical resistivity (10^8^–10^10^ Ω·cm) along with low dielectric loss in the high-frequency region [4,5]. The electrical, magnetic, and dielectric properties of spinel ferrites are highly sensitive to fabrication methodology and conditions, chemical composition (i.e., the amount of impurities and additives), cation distribution, and microstructures (i.e., grain size and shape). Substitution of transition and diamagnetic metal ions in spinel ferrites is a common and efficient approach to improve the magnetization ability and frequency-dependent permittivity and permeability [6].

Mn-substituted Mg ferrite is one of the most popular mixed spinel ferrite materials. Mg-Mn ferrites are suitable for use in low-power microwave components and devices in the X-band frequency region (8–12 GHz) because of their high initial permeability, low loss tangent, relative high temperature sensitivity, and narrow magnetic-resonance linewidth (several Gauss) [4,7,8,9]. Consequently, this material system has gained popularity in microwave communication and radar system applications. The physical properties of Mg-Mn ferrites can be improved by incorporating suitable trivalent metal ions into the basic ferrite system [10,11]. Several studies have reported that substitution of indium ions into Mg-Mn ferrite can increase the initial permeability and saturation magnetization [11,12]. Our previous study demonstrated that polycrystalline Mg-Mn-In ferrite exhibits better magnetization, higher AC magnetic susceptibility, and a higher Q × f value at a frequency of 6.5 GHz than do the Mg-Mn and Mg-Mn-Al ferrites fabricated by solid-state reaction method [13]. Sharma et al. have reported that Mg-Zn-Mn ferrite nanoparticles prepared by co-precipitation have low tan δ losses, making them a potential candidate material for microwave device applications such as planar circulators [6].

Nanocrystalline Mg-based ferrite particles have been successfully synthesized previously with the combustion [14,15], micro-emulsion [16], sol-gel [17], co-precipitation [18,19], and hydrothermal synthesis methods [20,21,22]. Among those wet chemistry routes (preparation techniques), the hydrothermal method is an environmentally friendly route for the preparation of crystalline nanoparticles without subsequent high-temperature annealing processing, with better compositional homogeneity for the synthesis of multi-component functional oxide materials, and with control over particle morphology and size [22,23]. A number of researchers have studied the effects of substitution of impurity ions and preparation methods on the crystallographic and magnetic properties of Mg-based ferrite nanoparticles [6,12,15,18]. In this study, the potential use and application of three kinds of Mg-based ferrite nanoparticles synthesized by the hydrothermal method were studied through investigating their physical properties, including crystal structure, crystallinity, phase purity, particle morphology, magnetic parameters, and microwave properties.

## 2. Materials and Methods

### 2.1. Preparation of Mg-Based Ferrite Nanoparticles

Mg-based ferrite nanoparticles with formulas of MgFe_2_O_4_, Mg_0.9_Mn_0.1_Fe_2_O_4_, and Mg_0.9_Mn_0.1_In_0.1_Fe_1.9_O_4_ were synthesized via hydrothermal synthesis from mixtures of metal nitrate hydrate solutions. Analytical grade metal nitrate hydrates, including magnesium nitrate hexahydrate, manganese (II) nitrate hexahydrate, indium (III) nitrate hydrate, and iron (III) nitrate nonahydrate (Alfa Aesar), were used as raw materials. Stoichiometric amounts of analytical grade reagents were dissolved in deionized (DI) water (the metal ion concentrations in the aqueous solutions were maintained at 0.05 M), and then sodium hydroxide solution was dropped into the resultant solutions until the pH value was 12. The precipitate was washed several times with DI water until a neutral pH value was obtained. After that, the suspension solution was poured into a 45-mL Teflon-lined stainless steel high-pressure autoclave reactor (Parr Instrument Company, Model 4744, Moline, IL, USA) for hydrothermal synthesis performed at 150 °C for 18 h. Finally, the products were centrifuged, washed, and dried to obtain the Mg-based ferrite nanoparticles.

### 2.2. Characterization of Ferrtie Nanoparticles

The crystal structure and phase purity of the synthesized Mg-based ferrite nanoparticles were analyzed with an X-ray diffractometer (XRD, Bruker D2 phaser, Karlsruhe, Germany) with Cu Kα radiation (λ = 1.5406 Å). Raman spectra were detected with a Triax 550 Raman spectrometer (Horiba Scientific, Edison, NJ, USA) using a 488 nm Ar-ion laser as the excitation source. The morphology and dimensions of the ferrite particles were analyzed with a high-resolution transmission electron microscope (HR-TEM, JEOL JEM2100F, Tokyo, Japan) operating at an accelerated voltage of 200 kV. Before the TEM observation, the obtained ferrite powders were dispersed in amyl acetate by ultrasonic oscillation, and then the nanoparticle suspensions were dropped onto copper grids coated with carbon film. Magnetization versus magnetic field (M-H) loops were examined using the vibrating sample magnetometry setting (VSM, Quantum Design MPMS 3, San Diego, CA, USA) in a external magnetic field of ±10 kOe. Alternating-current (AC) magnetic susceptibility (χ) measurements were performed as a function of frequencies (400 Hz–2.4 × 10^4^ Hz) by susceptibility analyzer (MagQu, XacQau, New Taipei, Taiwan). Magnetic resonance spectra were recorded on an electron paramagnetic resonance (EPR) spectrometer (Bruker EMX-10, Karlsruhe, Germany) at a frequency of about 9.86 GHz for evaluation of the microwave properties of the Mg-based nanoferrites. In the present study, the physical properties and characteristics of those ferrite nanoparticles were examined at room temperature (RT).

## 3. Results and Discussion

### 3.1. Structural and Morphological Characterization

**Figure 1:**
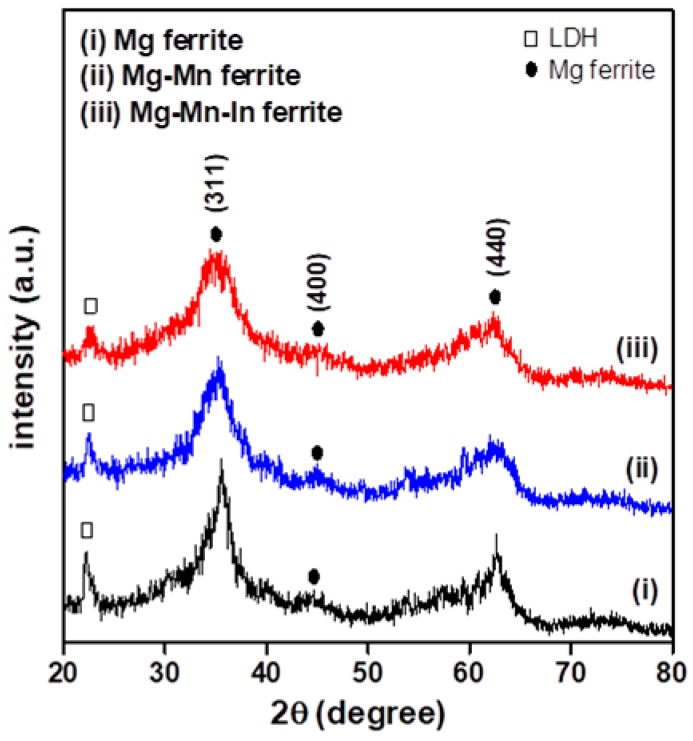
X-ray diffraction (XRD) patterns of Mg, Mg-Mn, and Mg-Mn-In ferrite nanoparticles synthesized by hydrothermal method.

The crystal structure and crystallinity of the obtained Mg-based ferrite particles were characterized by X-ray diffraction and Raman spectroscopy. Figure 1 presents the XRD patterns for the synthesized MgFe_2_O_4_ (Mg ferrite), Mg_0.9_Mn_0.1_Fe_2_O_4_ (Mg-Mn ferrite), and Mg_0.9_Mn_0.1_In_0.1_Fe_1.9_O_4_ (Mg-Mn-In ferrite) particles collected in the 2θ range from 20° to 80°. The significant background noise and broadening of the diffraction peaks were characteristic of crystalline metal oxide particles with nanometer dimensions [19,24]. The position (diffraction angle) and relative intensity of the diffraction peaks were compared with the standard powder X-ray diffraction data (JCPDS 17-0464) to examine the crystal structure of the ferrite particles. The synthesized ferrite powders were in the crystalline state, and two major and one weak diffraction peaks of XRD patterns corresponded to Bragg reflections of the (311), (440), and (400) planes of the cubic spinel structure (designated •). The full widths at half-maximum (FWHMs) of the (311) peaks for Mg, Mg-Mn, and Mg-Mn-In nanoferrites were 1.69°, 2.76°, and 3.72°, respectively. It is well known that the diffraction peak broadening of crystalline oxide materials is related to crystallite size reduction. The average nanocrystallite sizes of the three kinds of Mg-based ferrite particles were 5.8, 3.5 and 2.6 nm, which were estimated from XRD data using Scherrer’s formula (t = 0.9λ/Bcos × θ_B_, where t is the crystallite size, λ is the wavelength of the radiation, and θ_B_ and B represent the Bragg’s angle and the FWHM of the considered diffraction peak) [25].

It was found that incorporating Mn ions into the Mg ferrite reduced the average crystallite size, and the Mn-In co-substituted Mg ferrite exhibited the finest average crystallite size. That reduction can be explained by the ionic radius difference between Mg^2+^ (0.65 Å), Mn^2+^ (0.75 Å), In^3+^ (0.80 Å), and Fe^3+^ (0.64 Å) [26], which cause lattice distortion and change the nucleation rate. In addition, slight shifts in the diffraction peak of the (311) plane toward the low diffraction angle region were observed in the Mn-substituted and Mn-In co-substituted ferrite samples. That feature is related to the strain-induced distortion of the spinel crystal resulting from the incorporation of Mn and In into the MgFe_2_O_4_ nanoparticles. Figure 1 also shows the formation of the Mg nanoferrites accompanied by a small amount of layered double hydroxides (LDH, designated ⎕). This residual impurity disappeared after heat treatment of the hydrothermally synthesized products at 500 °C for 2 h under an air atmospere (Figure S1).

**Figure 2:**
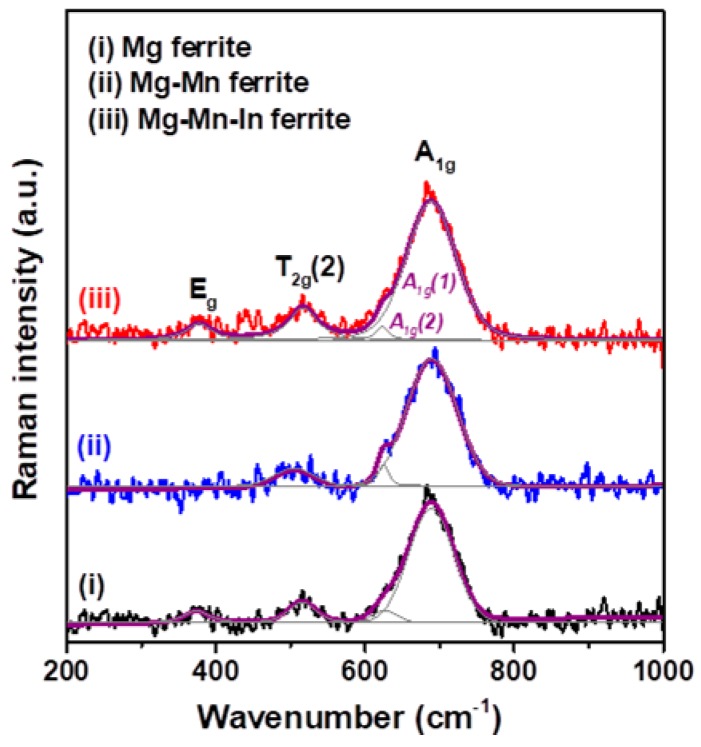
Raman spectra as a function of wavenumber for as-prepared Mg, Mg-Mn, and Mg-Mn-In nanoferrites.

Raman spectroscopy analysis is a powerful spectroscopic technique for revealing vibrational properties and providing critical information about similar structures [27]. For the cubic spinel structure, theory predicts that Raman active modes pertain to the motion of oxygen ions and both the tetrahedral A-site and octahedral B-site metal ions [20]. Recorded Raman spectra for the three Mg-based nanoferrites within the range of 200–1000 cm^−1^ are shown in Figure 2. Three Raman active modes were detected from the obtained Mg-based ferrite nanoparticles. They corresponded to the A_1g_, T_2g_(2) and E_g_ modes associated with the spinel structure and confirmed the formation of Mg ferrite. The presence of a strong Raman band in the 660–720 cm^−1^ region for cubic ferrites is related to the local lattice effect in the tetrahedral Fe^3+^O_4_ sublattice, whereas the Raman modes detected in the frequency of 460–640 cm^−1^ are related to the stretching vibration associated with the octahedral Fe^3+^O_6_ sublattice [28,29]. Moreover, as reported by Yan et al., the A_1g_ mode is ascribed to symmetric stretching of the oxygen anion, the T_2g_ mode is related to asymmetric stretching of the oxygen anion with respect to the A-site and B-site metal cations, and the E_g_ mode is due to symmetric bending of the oxygen anion [20]. In addition, Nakagomi et al. reported that the large mass difference between the Fe^3+^ and Mg^2+^ ions could split the A_1g_ mode into two branches [29]. The light Mg^2+^ ion corresponds to the Raman mode in the higher wavenumber region and is denoted A_1g_(1); the heavy Fe^3+^ ion corresponds to the Raman mode in the lower wavenumber region and is denoted A_1g_(2), as indicated in spectrum (iii) of Figure 2.

**Figure 3:**
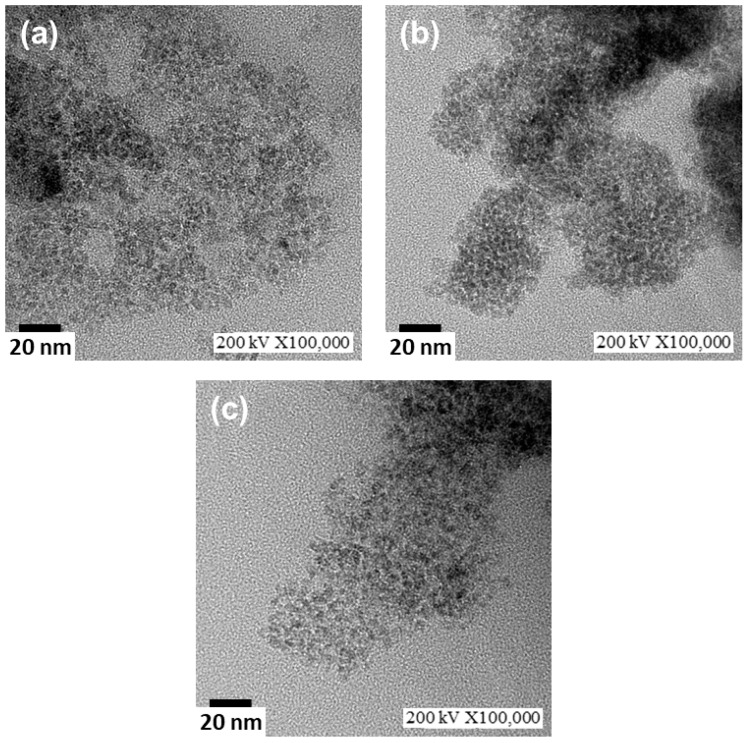
High-resolution transmission electron microscopy (HR-TEM) micrographs of Mg-based nanoferrites captured at a high magnification: (**a**) Mg, (**b**) Mg-Mn, and (**c**) Mg-Mn-In ferrite nanoparticles.

The morphology and electron diffraction of the three kinds of Mg-based ferrite nanoparticles were investigated using a JEOL JEM2100F HR-TEM. Figure 3 displays HR-TEM micrographs (bright field images) of the selected ferrite nanoparticles. In that figure, the ultra-fine ferrite particles are regular in shape, and they are agglomerated to some extent. This agglomeration is attributed to van der Waals forces and the single domain of the small ferrite particles; hence, each nanoparticle is permanently magnetized [17]. In addition, nanoparticles have a large surface area/volume ratio and possess very high surface energy. To reduce the total surface energy, the nanoparticles tend to agglomerate. On the other hand, functional metal oxides with a cubic crystal structure are prone to growth and form a spherical shape to minimize the surface free energy [24]. The particle diameter of the population was determined using the linear intercept method, and the particle size was evaluated from HR-TEM micrographs. The particle sizes of the nanoferrite samples were homogeneously distributed, and the mean particle sizes of the Mg, Mg-Mn, and Mg-Mn-In nanoferrites were determined to be 4.0 ± 0.9, 3.4 ± 0.6, and 3.3 ± 0.6 nm, respectively. The dimensions of the particles from the statistical analysis of HR-TEM micrographs were close to the crystallite size obtained from XRD analysis. The electron diffraction (ED) patterns in the supplementary information (Figure S2) show bright rings corresponding to the results of XRD examination, indicating the nanocrystalline nature of oxide nanoparticles synthesized by hydrothermal method.

### 3.2. Magnetic and Microwave Proeprties Analysis

The magnetic characteristics of spinel ferrites depend on the magnetic interaction (namely super-exchange interaction) between metal ions with magnetic moments in the tetrahedral A-site and octahedral B-site [30]. The cation distributions and relative strengths of preferred occupation sites were obtained from the literature. For example, Fe^3+^ (5 μ_B_) and Mn^2+^ (4.5 μ_B_) ions randomly occupy A-sites and B-sites. Non-magnetic Mg^2+^ (0 μ_B_) ions strongly prefer to occupy B-sites and partially occupy A-sites. It has been reported that non-magnetic In^3+^ (0 μ_B_) ions preferentially occupy A-sites up to x = 0.10 in Mg-Mn nanoferrites, beyond which they migrate to occupy B-sites [31].

**Figure 4:**
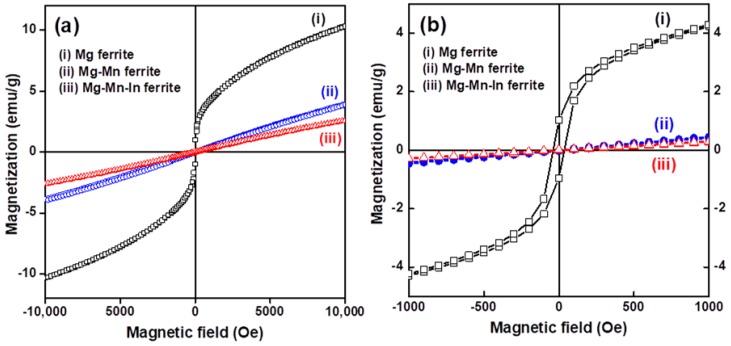
(**a**) Magnetization versus applied magnetic field for Mg, Mg-Mn, and Mg-Mn-In nanoferrites; (**b**) the magnetization (M-H) curves of the corresponding Mg-based spinel ferrite nanoparticles in the low magnetic field region (±1000 Oe).

Figure 4a depicts the magnetization of the three types of Mg-based ferrite nanoparticles as a function of the external magnetic field at RT. As clearly can be seen in those major M-H loops, the three Mg-based nanoferrites do not exhibit loss of magnetic hysteresis. In Figure 4b, the Mg nanoferrite shows weak hysteresis with a very small magnetic coercivity (37.8 Oe) at low magnetic field, which indicates superparamagnetic-like behavior. The value of magnetic coercivity of the obtained MgFe_2_O_4_ nanoparticles is smaller than that of the ZnFe_2_O_4_ (Hc = 67.3 Oe) and MnFe_2_O_4_ (Hc = 44.5 Oe) nanoparticles with sizes below 10 nm reported by Sabale et al. [32]. Superparamagnetic behavior can occur in nano-sized ferromagnetic and ferrimagnetic nanoparticles with a single domain because of weak interaction and thermal fluctuations of the spins of ferrite nanoparticles [33]. Pileni has described the thermal fluctuation effect on flips of spins between the easy magnetization axes, which lead to very small or negligible coercivity and remanence [34]. According to the Stoner-Wohlfarth theory, the magnetocrystalline anisotropy (E_A_) of a single-domain ferrite particle is expressed as follows [35]:

(1) $$E_{A}={\mathrm{KVsin}}^{2}\theta$$

where K is the magnetocrystalline anisotropy constant, V is the size of the ferrite nanoparticle, and θ is the angle between the magnetization direction and easy axis of the ferrite nanoparticle. The major factors in the control or formation of superparamagnetism are magnetocrystalline anisotropy and the dimension (size) of ferrite nanoparticles [18]. Chen et al. attributed the superparamagnetic properties of MgFe_2_O_4_ nanoparticles to the size dependence of the magnetocrystalline anisotropy in the ferrite nanoparticles [36]. Modi et al. reported that when the blocking temperature of the ferrimagnetic nanoparticles is lower than RT, the ferrite nanoparticles should exhibit a superparamagnetic nature [19]. Above the blocking temperature, the magnetocrystalline anisotropy can be overcome by thermal activation; therefore, the magnetocrystalline anisotropy constant may approach zero and the magnetization direction of nanoferrites can easily follow the direction of the external magnetic field. The Stoner–Wohlfarth theory also describes the relationship between the coercivity (Hc) and magnetocrystalline anisotropy constant (K). Since Hc is proportional to K (Hc ∝ K) and the value of K is near zero, the obtained Mg nanoferrites had relatively small values of the coercive field.

Figure 4 also shows that the magnitude of magnetization for both Mg-Mn and Mg-Mn-In nanoferrites increased with increases in magnetic field, and the two impurity-substituted Mg-nanoferrites exhibited a significant paramagnetic nature. A previous study reported that Mg ferrite nanocrystallites smaller than 8 nm, achieved by co-precipitation method, could exhibit paramagnetic behavior [36]. The single-domain behavior of the nano-sized ferrite particles, which have a paramagnetic nature, may be the result of the low number of collinear spins in those particles [21]. It is noted that the Mg nanoferrite sample did not reach complete saturation even under a high magnetic field of 10 kOe (Figure 4a). This feature is often observed in spinel ferrite nanoparticles. Several authors have reported the reduction of magnetization in nanoparticles and proposed mechanisms to explain the no-saturation behavior in a high magnetic field. It can be attributed to the presence of a spin disordered surface layer, which requires a larger magnetic field to reach saturation magnetization [37]. The saturation magnetization (estimated from the linear extrapolation of M vs. 1/H plot) of the Mg nanoferrite sample was found to be 12.8 emu/g (Figure S3). Table 1 summarizes the magnetic parameters and microwave properties of three Mg-based nanoferrites. The chemical composition can significantly influence the magnetization behavior because of changes in the distribution of cations and the particle size. The reduction of the magnitude of magnetization is ascribed to a decrease in the ferrite nanoparticle size because of the noncollinear spin arrangement at the particle surface and the difference in the magnetization characteristic of two sub-lattices due to cation redistribution [38]. The disordered or misaligned surface spins weaken the total magnetization of the ferrite nanoparticles. Due to the surface spins of structural distortion and redistribution of the cations to less-preferred sites, the magnitude of magnetization of Mg-based nanoferrites is lower than that of bulk ones [13]. Mohseni et al. explained that the angular momentum of Mn^2+^ ions is zero, which can reduce the coercivity of Mg ferrites [38]. Moreover, a decrease in saturation magnetization has been reported to result from partial replacement of Fe^3+^ by In_3_^+^ ions in Mg-Mn-Ni ferrites when the In^3+^:Fe^3+^ ratio is 0.1:1.9 [31].

**Figure 5:**
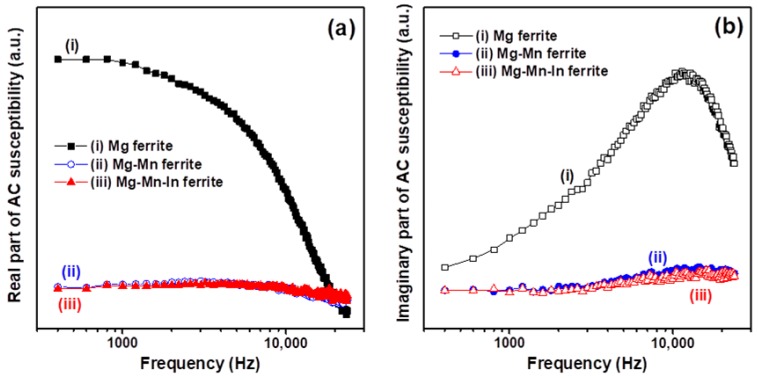
Frequency-dependent alternating-current (AC) magnetic susceptibility (χ) spectra of Mg, Mg-Mn, and Mg-Mn-In nanaferrites: (**a**) real part (χ′) and (**b**) imaginary part (χ″).

Figure 5 depicts alternating current (AC) magnetic susceptibility spectra for the three types of Mg-based ferrite nanoparticles investigated in this study. The AC magnetic susceptibility of magnetic materials includes two parts: the real component (χ′) and the imaginary component (χ″). The magnetization of Mg nanoferrites is higher than that of the Mg-Mn and Mn-Mn-In nanoferrites discussed above (see Figure 4a). In addition, the real component (χ′) is the slope of the M-H curve in the limit of low frequency. Thus, the magnitude of the real part of the complex susceptibility for the un-substituted nanoferrite sample is significantly greater than that of the Mn substituted and Mn-In co-substituted nanoferrite samples, as shown in Figure 5a. Moreover, the magnitude of the real part of the susceptibility for the Mg-based nanoferrites decreases as the driving frequency approaches a constant value. This decrease is related to the rotation speed of the magnetic moment lagging behind the driving frequency. The imaginary part of the complex susceptibility disclosed a dissipative process (namely magnetic loss) in the magnetic oxide nanoparticles during AC magnetic measurement. The characteristic resonance frequency of the Mg nanoferrites (spectrum (i) in Figure 5b) is 12 kHz. Since the magnetization ability of the Mn-substituted and Mn-In co-substituted Mg nanoferrites is weaker than that of the un-substituted Mg nanoferrite, the magnitude of the imaginary component (χ″) of the former two is lower than the latter and accompanied by a reduction of the magnetic loss peak (Figure 5b). In addition, we found that the resonance frequency of the hydrothermal synthesized Mg nanoferrite was close to 12 kHz, which is very close to the resonance frequency of the Mg ferrite prepared via solid state method [13].

**Figure 6:**
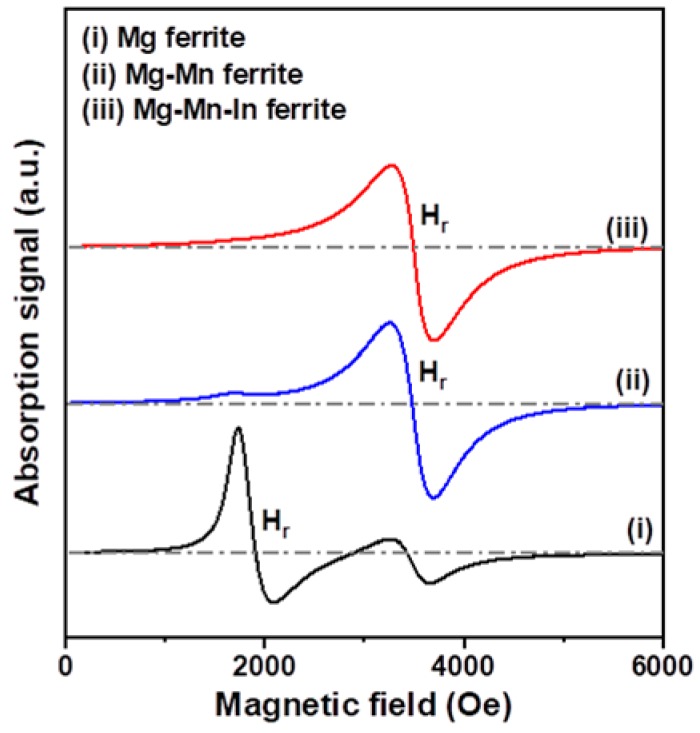
Comparison of X-band magnetic resonance spectra of Mg, Mg-Mn, and Mg-Mn-In nanoferrites.

In this study, the microwave properties of Mg-based nanoferrites were investigated by electron paramagnetic resonance (EPR) spectroscopy. We determined the magnetic resonance field (Hr) and resonance linewidth (ΔH) from each magnetic resonance spectrum to understand the magnetic dipolar interaction and super-exchange interaction [39]. The value of the Landé g-factor can be calculated by using the following Equation:

(2) $$h\upsilon =g\mu_{B}B_{0}$$

where h is Planck’s constant, ν is a microwave frequency, μ_B_ is the Bohr magnetron, and B_0_ is an applied magnetic field. Investigation of EPR for Mg-based nanoferrites was performed at a microwave frequency of 9.86 GHz. The EPR spectra of the three nanoferrite samples are plotted in Figure 6, and the obtained resonance linewidth (ΔH), resonance field (Hr), and Landé g-factor are given in Table 1. Guskos et al. reported that the linewidths of ZnFe_2_O_4_ nanoparticles prepared by wet chemistry method are 450–750 Oe [40]. They suggested that the magnetic resonance feature is strongly affected by the slightly asymmetrical and very intense broad line. Thota et al. reported that the linewidths of citrate-gel synthesized (Mn_x_Zn_1−x_)Fe_2_O_4_ nanoparticles increase from 309 to 660 Oe when the Mn content is increased from x = 0.35 to x = 0.65 [41]. In the present case, the resonance linewidth of Mg-based ferrite nanoparticles was 350–440 Oe, and the linewidth of the un-substituted sample was narrower than those of the Mn substituted and Mn-In co-substituted samples. The resonance linewidth can be used for evaluating the magnetic field homogeneity in ferrite nanoparticles. Therefore, the narrower resonance linewidth of the ferrite samples indicates better homogeneity [41]. It can be mentioned that substitution of Mn into Mg nanoferrite weakens the super exchange interaction and broadens the resonance linewidth. We also found that the resonance field of the Mg-based nanoferrite significantly shifted from 1910 Oe to almost 3500 Oe after Mn and Mn-In were incorporated into Mg nanoferrites and that the g-factor values decreased from 3.690 to about 2.020 because the g-factor is inversely proportional to the resonance field, according to Equation (2). It is reported that ferrite samples exhibit a low resonance field due to crystalline anisotropy [42].

## 4. Conclusions

Nano-sized Mg-based ferrites were successfully synthesized by hydrothermal method at a low temperature of 150 °C. XRD examination and Raman spectroscopy analysis confirmed the formation of the cubic spinel phase of Mg-based nanoferrites. XRD examination and TEM observation showed that the particle sizes of un-substituted Mg nanoferrites were greater than those of Mn substituted and Mn-In co-substituted ones. Magnetic property measurements indicated that the Mg nanoferrite had the best magnetization ability, the Mg nanoferrite exhibited superparamagnetic behavior, and both the Mg-Mn and Mg-Mn-In nanoferrites displayed paramagnetic natures. In this study, the Mg nanoferrite had the highest real part of AC magnetic susceptibility and the narrowest resonance linewidth of 355 Oe at a frequency of 9.86 GHz.

## Figures and Tables

**Table 1:** Magnetic parameters and microwave properties of three Mg-based nanoferrites.

Nanoferrite Sample	M (emu/g)	Hc (Oe)	Mr (emu/g)	ΔH (Oe)	Hr (Oe)	g-Factor
Mg	10.36	37.8	1.0	355	1910	3.690
Mg-Mn	3.97	-	-	440	3480	2.024
Mg-Mn-In	2.63	-	-	420	3495	2.016

M: Magnetization at 10 kOe applied magnetic field; Hc: coercivity; Mr: remanence; ΔH: resonance linewidth; Hr: resonance field; g: Landé g-factor.

## Supplementary Materials

The following is available online at http://www.mdpi.com/1996-1944/11/11/2274/s1, Figure S1: X-ray diffraction (XRD) patterns of Mg, Mg-Mn, and Mg-Mn-In ferrite nanoparticles after annealing at 500 °C for 2 h, Figure S2: Electron diffraction (ED) patterns of Mg-based nanoferrites: (a) Mg, (b) Mg-Mn, and (c) Mg-Mn-In ferrite nanoparticles, Figure S3: Plot of magnetization (M) versus the reciprocal of the magnetic field (1/H) for Mg ferrite nanoparticles in the high field.
