# Supplementary material for: Hydrothermally Synthesized Mg-Based Spinel Nanoferrites: Phase Formation and Study on Magnetic Features and Microwave Characteristics
Source: Materials (Basel). 2018 Nov 14;11(11):2274. doi: 10.3390/ma11112274 (PMC6266840; doi:10.3390/ma11112274)
Supplement: Supplementary file 1 [file materials-11-02274-s001.pdf]

materials

MDPI

Article

Hydrothermally Synthesized Mg-based Spinel Nanoferrites: Phase Formation and Study on Magnetic Features and Microwave Characteristics

Chien-Yie Tsay [1], Yi-Chun Chiu [1], Chien-Ming Lei [2]

[1] Department of Materials Science and Engineering, Feng Chia University, Taichung 40724, Taiwan; a0928402248@gmail.com
[2] Department of Chemical and Materials Engineering, Chinese Culture University, Taipei 11114, Taiwan; ljm9@faculty.pccu.edu.tw
* Correspondence: cytsay@mail.fcu.edu.tw; Tel.: +886-4-2451-7250 (ext. 5312); Fax: +886-4-2451-0014

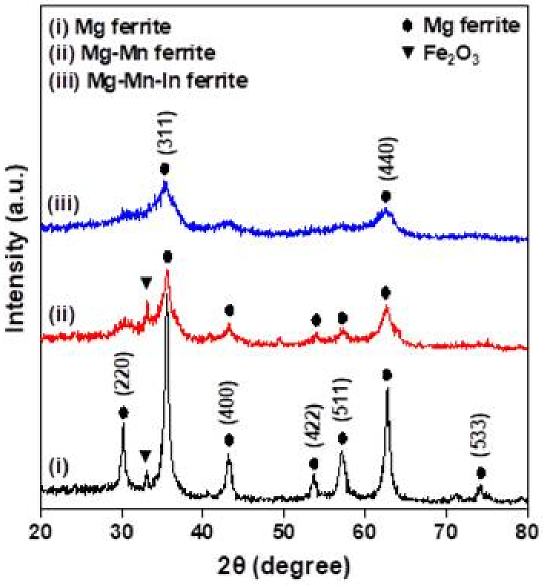

Figure S1. X-ray diffraction (XRD) patterns of Mg, Mg-Mn, and Mg-Mn-In ferrite nanoparticles after annealing at 500 °C for 2 h.

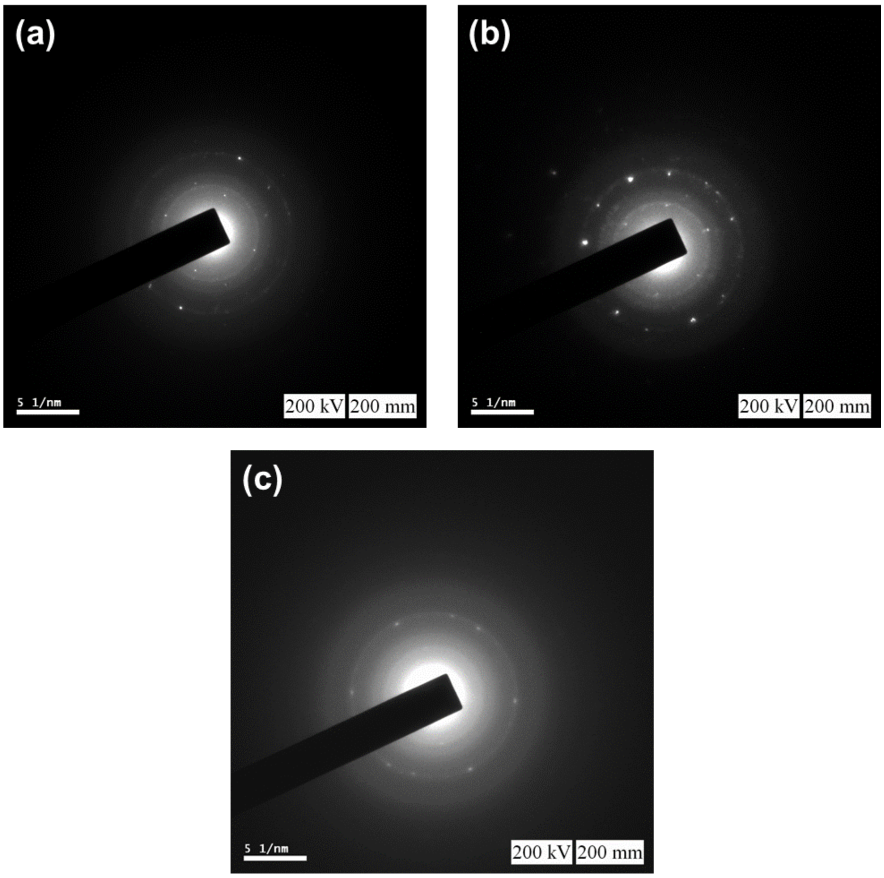

Figure S2. Electron diffraction (ED) patterns of Mg-based nanoferrites: (**a**) Mg, (**b**) Mg-Mn, and (**c**) Mg-Mn-In ferrite nanoparticles.

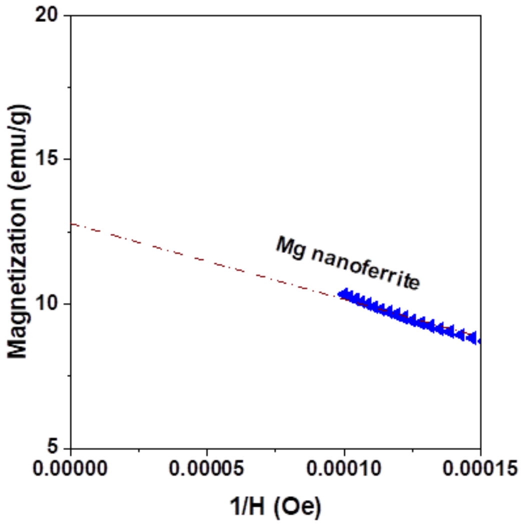

Figure S3. Plot of magnetization (M) versus the reciprocal of the magnetic field (1/H) for Mg ferrite nanoparticles in the high field.

© 2018 by the authors. Licensee MDPI, Basel, Switzerland. This article is an open access article distributed under the terms and conditions of the Creative Commons Attribution (CC BY) license (http://creativecommons.org/licenses/by/4.0/).
